# Evaluation of Flexural Resistance in Co-Cr Ceramic Systems: Conventional Casting Versus 3D Printing—A Pilot Study

**DOI:** 10.3390/dj13120583

**Published:** 2025-12-05

**Authors:** Alexandra Elena Biculescu, Anca Iuliana Popescu, Tudor-Petru Ionescu, Ioana Ana Maria Ciorniciuc, Daniel Alexandru Referendaru, Costin Coman, Andrei Constantinovici, Stefan-Eugen Chirsanov-Capanu, Mirel Stoian-Albulescu, Raluca Monica Comaneanu

**Affiliations:** 1 Doctoral School of Dental Medicine, Titu Maiorescu University, 189 Calea Văcărești, 040051 Bucharest, Romania; 2 Faculty of Dental Medicine, Titu Maiorescu University, No. 67A, Gheorghe Petrașcu Street, 031593 Bucharest, Romania

**Keywords:** cobalt-chromium alloy, metal-ceramic bond strength, flexural resistance, selective laser melting, additive manufacturing, surface treatment, sandblasting, dental ceramics

## Abstract

**Introduction**: The success of metal–ceramic restorations depends on the mechanical and adhesive properties of the metal–ceramic interface. With the emergence of additive manufacturing technologies such as selective laser melting (SLM), there is growing interest in comparing these methods with conventional casting. This pilot study aimed to generate hypothesis-forming data on how fabrication method (casting and 3D printing) and alumina sandblasting with two particle sizes (125 μm and 250 μm) influence flexural performance of Co-Cr metal–ceramic systems within the standardized ISO 9693 framework. **Materials and Methods**: Rectangular Co-Cr alloy specimens were manufactured using two techniques: conventional casting and 3D printing via SLM. Each group was divided based on the sandblasting particle size. After ceramic application in accordance with ISO 9693:2012, samples underwent a three-point bending test using a universal testing machine (Instron 8872) to assess the displacement force required to fracture the ceramic layer. Five specimens were tested per group, and mean values and standard deviations were calculated. Data were statistically analyzed using two-way ANOVA followed by Tukey’s HSD post hoc test (*p* < 0.05). **Results**: Cast samples exhibited significantly higher displacement strength than printed ones. Among all groups, the cast samples sandblasted with 250 μm particles (CCT_250) showed the best performance (mean: 12.48 ± 0.91 N), while the 3D-printed group treated with 125 μm particles (CCP_125) showed the lowest strength (mean: 7.24 ± 0.65 N). Larger abrasive particles (250 μm) improved bond strength in both fabrication techniques. Two-way ANOVA revealed significant main effects of manufacturing method (F(1,16) = 13.63, *p* = 0.002, η^2^ = 0.46) and particle size (F(1,16) = 6.17, *p* = 0.024, η^2^ = 0.28), with no interaction between factors. **Conclusions**: Both the manufacturing method and the sandblasting protocol significantly influence the flexural performance of Co-Cr ceramic systems. Conventional casting combined with 250 μm particle sandblasting ensures the highest ceramic adhesion, while SLM-printed substrates may require additional surface treatments to improve bonding efficiency. Complementary surface treatments such as bonding agents or chemical oxidation may enhance the metal–ceramic bond in SLM-fabricated frameworks.

## 1. Introduction

The bond between the Co-Cr framework and the ceramic layer is a critical factor in the success of metal–ceramic restorations used in daily prosthetic practice. The durability of the metal–ceramic contact has a direct effect on how long the restoration lasts and how well it resists chipping or delamination [1]. Despite the advent of all-ceramic materials, metal–ceramic restorations remain widely used, especially in posterior areas, due to their robustness, strength, and long-term stable clinical behavior [2,3]. The cobalt–chromium alloys used for fixed prosthetic restorations have been thoroughly examined from multiple perspectives, including biocompatibility, corrosion resistance, microstructural integrity, and mechanical performance under simulated clinical conditions [4,5,6,7,8,9,10,11].

Cobalt–chromium (Co-Cr) alloys have gained increasing popularity in this context due to their favorable combination of mechanical strength, biocompatibility, and low cost. In addition, these alloys exhibit good corrosion resistance and dimensional stability, making them suitable for extensive prosthetic frameworks [12].

The evolution of manufacturing technologies has allowed the transition from conventional casting methods to modern additive manufacturing techniques, such as selective laser melting (SLM), which allows the production of precise structures with minimal material loss [13]. However, the microstructure and surface characteristics of 3D-printed parts differ from cast parts, which may influence the metal–ceramic interface and, implicitly, the clinical behavior of the restorations [14,15].

The roughness of the metal surface plays an essential role in the mechanical anchorage of the ceramic and in the development of an effective adhesion. The abrasive particle blasting process is frequently used to improve surface properties [16]. It has been shown that the size of the abrasive particles affects the adhesion of the ceramic, with the optimal values being still intensively investigated [17,18,19].

In this context, the aim of the present pilot study is to generate hypothesis-forming data on how the manufacturing method (casting vs. three-dimensional printing) and the two-dimensional aluminum oxide blasting treatment (125 μm and 250 μm) influence the adhesion of ceramics applied to Co-Cr alloys within the standardized ISO 9693 framework [14,20]. The test method used is the three-point mechanical bending test, according to the EN ISO 9693:2012 standard, recognized for its reproducibility and relevance in metal–ceramic adhesion studies [21].

## 2. Materials and Methods

This study was conducted as an exploratory pilot investigation to analyze trends between production methods and particle size under ISO 9693 three-point bending conditions. Each group had n = 5 specimens, a sample size frequently employed in pilot bench-top investigations to assess effect sizes and variation for powering further confirmatory experiments [22]. Due to the exploratory nature, we emphasized estimating (effect sizes and confidence intervals) rather than hypothesis testing.

To investigate the bending adhesion behavior of metal–ceramic systems, experimental samples were made in the form of rectangular Co-Cr alloy plates, manufactured by two different technologies: conventional casting and 3D-printing by selective laser melting (selective laser melting—SLM), a methodology widely used in adhesion testing of metal–ceramic systems [23]. Subsequently, these plates were subjected to a standardized ceramic application protocol, according to the EN ISO 9693:2012 standard, and mechanically tested by the three-point bending method [24,25]. Co–Cr plates were produced using a commercial selective laser melting (SLM), in an inert argon environment using a TRUMPF TruPrint 1000 machine (TRUMPF TruPrint 1000 machine (TRUMPF GmbH + Co. KG, Ditzingen, Germany)) by a recognized dental manufacturing center. The manufacturer’s technical literature states that the system utilizes a single-mode TRUMPF fiber laser with a maximum power of 200 W and a beam width of 80 µm (standard optic), with a configurable layer thickness range of 20–60 µm. In the current fabrication run, the operator employed process settings optimized for dental Co-Cr alloys, which include the following:

- Laser power: 160–195 W;
- Scan speed: 600–1200 mm/s;
- Layer thickness: 30 µm;
- Hatch spacing: 90–120 µm;
- Scan strategy: Island scanning with 3–5 mm tiles, combined with a meander pattern and rotation of the scan vector between layers (±67°) to reduce residual stresses and anisotropy;
- Build atmosphere: High-purity argon (O_2_ < 0.1%).

The alloy employed was Mediloy S-Co (BEGO GmbH, Bremen, Germany), a Co-Cr-W-Mo powder approved for SLM applications (grain size 10–45 µm), compliant with ISO 22674 and ISO 9693. The manufacturer’s information provides typical SLM operational parameters for this material, including a laser power range of 95–195 W, a scan speed of 1120–1400 mm/s, and a powder layer thickness of 30 µm, which align with those employed in this investigation.

Following production, all SLM frameworks were subjected to a standardized post-processing stress-relief heat treatment at 800 °C, adhering to BEGO’s prescribed cycle (ramp from 650 °C to 800 °C in 12 min, hold for 15 min, followed by controlled cooling to 550 °C).

All specimens were cleansed, de-powdered, and detached from the assembly platform in accordance with the manufacturer’s methodology.

**Figure 1:**
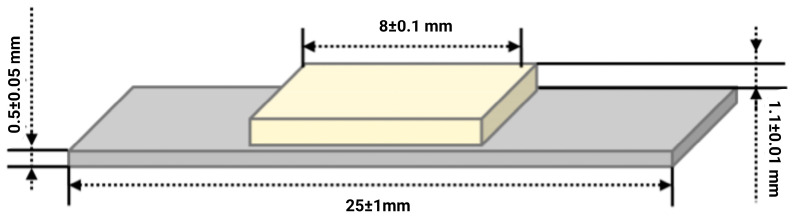
Dimensional requirements imposed on experimental samples according to ISO 9693:2012 standard for performing the three-point bending test.

According to this standard, the experimental samples subjected to the three-point bending mechanical test, to test the adhesion of ceramics to metal, must meet the dimensional requirements illustrated in Figure 1. Similar geometries have been employed by several studies assessing Co–Cr and Ti alloys to ensure standardized stress distribution [18].

### 2.1. Preparation of Metal Samples

The metal samples were made in four experimental variants, depending on the manufacturing method and the size of the abrasive particles used for sandblasting. The coding of the samples is presented in Table 1.

Sandblasting was performed using aluminum oxide (Al_2_O_3_) particles of two grain sizes—125 µm and 250 µm—applied uniformly to all specimens according to ISO 9693:2012 recommendations. The procedure aimed to optimize surface roughness and improve ceramic adhesion. The exact blasting parameters (pressure, distance, and duration) were not documented in the original laboratory protocol [16,22].

### 2.2. Application of the Ceramic Layer

**Figure 2:**
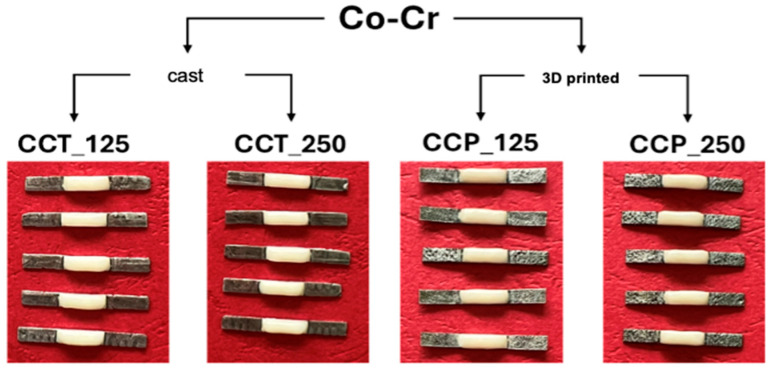
Appearance of samples for three-point bending mechanical tests.

The deposition of the ceramic layer was carried out in three successive stages, according to the recommendations of the ISO 9693:2012 standard and the guidelines provided by the manufacturer of the dental ceramics used (Figure 2) [15]:

1. Application of opaque layer 1: A paste obtained by mixing opaque powder with liquid was distributed homogeneously over approximately 70% of the surface of the plate. The sample was placed in the ceramic furnace and fired at a temperature of 970 °C.
2. Application of opaque layer 2: A complete coverage of the surface was achieved with a second opaque layer, giving a slightly cream tint after firing.
3. Dentin deposition: A uniform layer of dentin was applied to a thickness of (1.1 ± 0.1) mm using a calibrated template, and the sample was sintered according to the thermal curve recommended by the manufacturer. According to ISO 9693:2012, the ceramic layer was applied symmetrically over a length of 8.0 ± 0.1 mm on one side of the plate. This method is considered one of the most appropriate for evaluating the adhesion between dental metal and ceramic materials, as the fabrication of experimental samples is relatively simple and the tests are reproducible [26].

### 2.3. Three-Point Mechanical Bending Test

**Figure 3:**
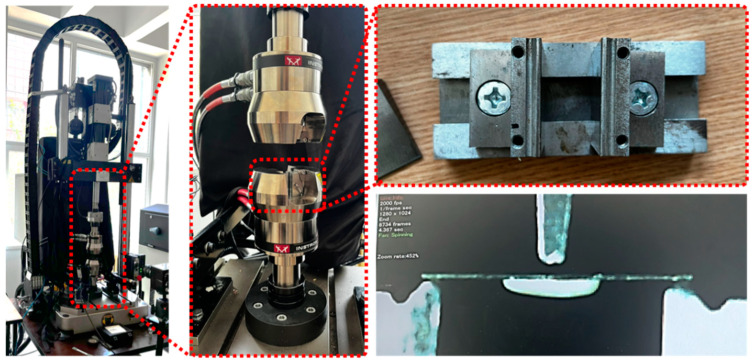
Instron 8872 mechanical testing machine, support clamps used in three-point bending tests, and detailed image of a test being performed.

The evaluation of the displacement strength of the ceramic layer was performed using a universal mechanical testing machine (Instron 8872, Instron, Norwood, MA, USA), according to the ISO 9693:2012 protocol [27]. The samples were mounted in a dedicated metal support, with the ceramic layer oriented on the side opposite to the force application, so that the adhesion of the metal–ceramic interface could be directly evaluated (Figure 3).

### 2.4. Statistical Analysis

Statistical analysis was conducted utilizing JASP version 0.18 (JASP Team, University of Amsterdam, Amsterdam, The Netherlands). The homogeneity of variances was verified using Levene’s test, and the distribution of data was evaluated using the Shapiro–Wilk test. Given that all groups exhibited normal distribution (*p* > 0.05) and homogeneity of variances (*p* > 0.05), a two-way analysis of variance (ANOVA) was performed to assess the impacts of the manufacturing method (conventional casting versus selective laser melting—SLM) and particle size (125 µm versus 250 µm) on displacement force values.

Post hoc analyses were conducted with Tukey’s HSD test with a significance threshold of *p* < 0.05. Alongside the *p*-values, effect sizes (partial η^2^) were obtained to assess the contribution of each factor to the overall variance. Results are shown as mean ± standard deviation (SD) and 95% confidence intervals (CIs) for each experimental group.

## 3. Results

The three-point mechanical bending tests allowed the evaluation of the displacement strength of the ceramic layer applied to Co-Cr alloys, depending on the manufacturing method of the metallic substrate and the size of the abrasive particles used for sandblasting. For each experimental group, five values of the displacement strength, expressed in Newtons (Ns), were recorded, and the arithmetic mean and standard deviation were subsequently calculated. Additionally, we report 95% confidence intervals for each group.

### 3.1. Individual Results of the Bending Tests

Table 2 presents the values recorded for each sample, as well as the corresponding mean values and standard deviations.

Beyond descriptive statistics, estimation analysis showed clear ordering of group means: CCT_250 (11.35 N, 95% CI 9.01–13.69) > CCT_125 (9.95 N, 95% CI 8.76–11.14) > CCP_250 (9.19 N, 95% CI 7.63–10.74) > CCP_125 (7.45 N, 95% CI 5.73–9.17). Two-way ANOVA indicated significant main effects of the manufacturing method (F(1,16) = 13.63, *p* = 0.002, partial η^2^ = 0.46) and particle size (F(1,16) = 6.17, *p* = 0.024, partial η^2^ = 0.28), with no interaction (F(1,16) = 0.07, *p* = 0.79). Pairwise estimation (Welch) confirmed larger forces for CCT_250 vs. CCP_125 (+3.90 N, 95% CI +1.45 to +6.35) and for CCT_125 vs. CCP_125 (+2.50 N, 95% CI +0.73 to +4.27). Post hoc Tukey’s HSD indicated higher bond strength for CCT_250 compared with CCP_125 (*p* = 0.0024), while other pairwise differences were not statistically significant. Given the pilot sample size (n = 5/group), effect sizes (partial η^2^) and 95% confidence intervals were emphasized alongside *p*-values to aid interpretation.

**Figure 4:**
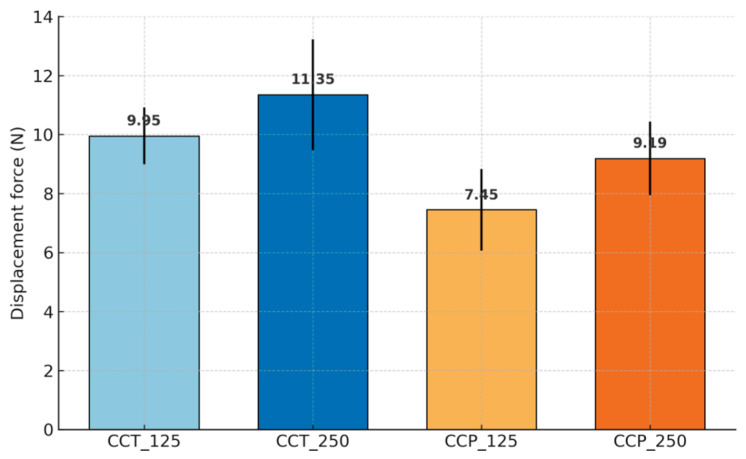
Evolution of average detachment forces for the investigated materials.

The 95% confidence intervals for mean displacement forces ranged from 5.73 to 9.17 N for CCP_125 and 9.01–13.69 N for CCT_250 (Table 2). The values clearly highlight a variability in the displacement force depending on the type of substrate and the surface treatment applied. This variation is also reflected in the graph in Figure 4, where the mean displacement force values for each experimental group are illustrated, along with the error bars corresponding to the standard deviations.

More consistent force–displacement patterns were seen in cast samples, which is suggestive of cohesive ceramic fracture and a stable oxide layer. In contrast, the SLM groups had irregular load responses and lower mean forces, aligning with less consistent oxide production and increased surface porosity, as shown in prior research [13,28].

### 3.2. Analysis of Force–Displacement Curves

The recordings made by the universal mechanical testing machine (Instron 8872) provided force–displacement curves for each sample. These provide additional information about the behavior of the samples under load, as well as about the failure mode of the ceramic–metal interface.

**Figure 5:**
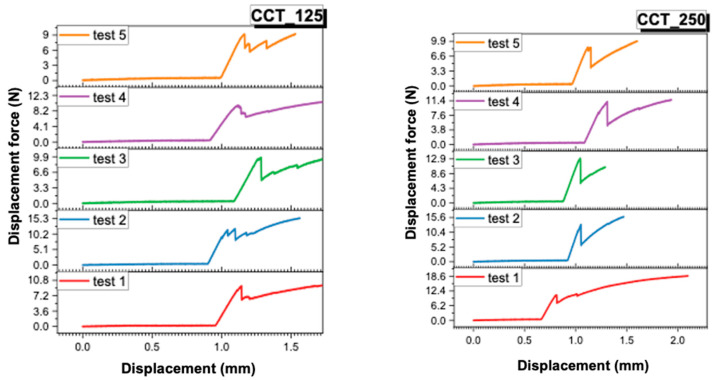
Force–displacement curves recorded for CCT_125 and CCT_250.

**Figure 6:**
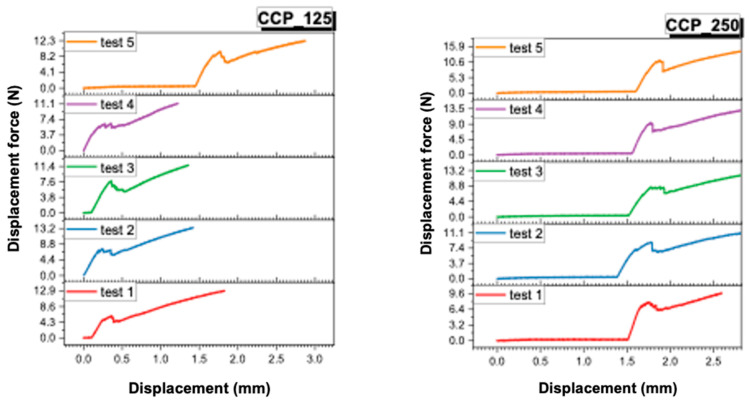
Force–displacement curves recorded for CCP_125 and CCP_250.

**Figure 7:**
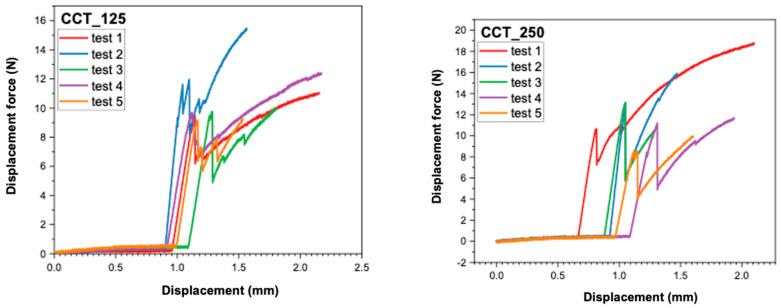
Superimposed force–displacement curves recorded for CCT_125 and CCT_250.

**Figure 8:**
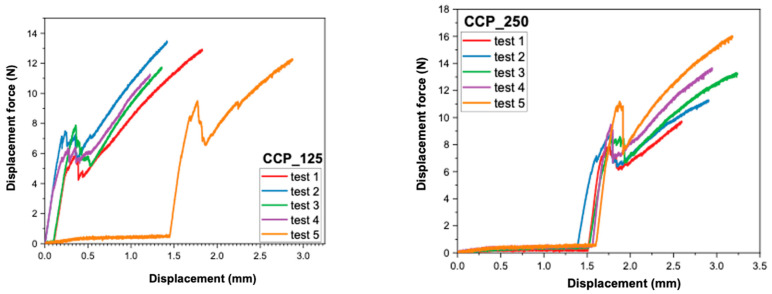
Superimposed force–displacement curves recorded for CCP_125 and CCP_250.

- In Figure 5 and Figure 6, the individual curves for each of the four material groups (CCT_125, CCT_250, CCP_125, CCP_250) are presented.
- In Figure 7 and Figure 8, the superimposed curves, per group, are illustrated for a visual comparison of the consistency of the mechanical behavior.

The force–displacement curves recorded for each group (CCT_125, CCT_250, CCP_125, and CCP_250) revealed distinct mechanical behaviors. Cast Co-Cr specimens (CCT groups) exhibited smoother and more reproducible curves with a gradual load increase and a well-defined peak, indicating stable adhesion and cohesive resistance of the veneering ceramic. In contrast, SLM samples (CCP groups) showed irregular or discontinuous profiles, often characterized by sudden drops in load, which may reflect a more fragile metal–ceramic interface and the presence of localized stress concentrations.

Although no direct visual examination of fracture surfaces was performed in this study, the differences observed in the curve profiles suggest a stronger and more consistent bond in cast specimens and a weaker, less predictable adhesion in 3D-printed ones. Future studies will include microscopic evaluation of fracture surfaces to confirm the correlation between curve morphology and failure mode (adhesive, cohesive, or mixed).

### 3.3. Comparative Observations

The analysis of the results allows the extraction of the following findings:

- The samples manufactured by casting (CCT) presented higher displacement forces than those obtained by 3D-printing (CCP), regardless of the size of the sandblasting particles.
- The larger size of the Al_2_O_3_ particles (250 μm) led to increased adhesion, for both the cast and printed samples.
- The best performance was obtained by the CCT_250 group, with an average displacement force value of 12.48 N (±0.91 N).
- The weakest adhesion was recorded for the CCP_125 group, with an average force of 7.24 N (±0.65 N).

## 4. Discussion

Analysis of the results reveals two major influences on the ceramic-to-metal bond strength: the CoCr alloy fabrication method and the Al_2_O_3_ particle size used in the sandblasting [27].

The cast samples (CCT) recorded higher values of bond strength compared to those obtained by SLM (CCP), as evidenced by the mean score of 12.48 N vs. 8.96 N for the 250 μm treatment. These results align with the findings of Hong et al. [27], and others [12], who demonstrated that the cast group had the highest metal–ceramic bond strength, although all methods exceeded the ISO standard thresholds [11]. In addition, the fine microstructure, layering and relatively high porosity of the SLM surfaces were associated with lower mechanical adhesion, as also observed by Revilla León et al. [13].

The use of 250 μm particles resulted in a higher level of displacement strength than 125 μm, consistent with the studies by Derand and Herø and Gołębiowski and Pietnicki [29], who reported maximum efficiency in surface treatment with particles around 110–250 μm for CoCr and Ti alloys [30]. Furthermore, the study by Yassin et al. [19] concluded that blasting with 250 μm Al_2_O_3_ produces increases in roughness and fracture toughness, compared to clinical grinding with smaller particles.

This pilot study did not include microscopic evaluation; however, comparable research by Łagodzińska et al. [31] and Hasan et al. [32] demonstrated that 250 µm sandblasting generates deeper surface asperities and a more retentive morphology than 125 µm, resulting in enhanced mechanical interlocking between ceramic and metal.

The results suggest that the manufacturing method influences both the surface morphology and the microstructure of the substrate—casting generates a more compact and less layered surface, favorable for mechanical adhesion. Blasting with large particles enhances this effect by increasing the depth of asperities and the active surface area.

In addition to the mechanical role of surface roughness, a significant factor affecting the link between metal and ceramic is the chemical adhesion facilitated by the oxide layer. This intermediate layer, which serves as a chemical link between the silica-rich vitreous phase of the veneering ceramic and the metallic substrate in Co–Cr systems, is mostly made up of chromium oxide (Cr_2_O_3_). The production technique significantly affects the composition, thickness, and uniformity of this oxide layer. Traditionally cast alloys, which become harder gradually, often provide a consistent and stable Cr_2_O_3_ coating, promoting reliable ceramic adhesion. In contrast, alloys produced using SLM solidify quickly under localized melting conditions, resulting in a finer grain structure, modified chromium distribution, and the possible development of mixed oxides (Cr_2_O_3_, MnO, SiO_2_) with varying thickness.

Recent studies have shown that post-processing parameters, including annealing temperature and regulated oxidation, significantly influence oxide morphology and metal–ceramic bond efficacy in SLM Co–Cr alloys, with annealing at around 1150 °C resulting in improved adhesion and more uniform interfacial properties [28]. The metallurgical structure produced by various production techniques (casting, milling, or laser melting) has been linked to differences in oxide film continuity and bond strength [33]. Furthermore, novel surface treatment techniques—such as refined sandblasting and oxidation methods—have demonstrated the capacity to improve oxide–ceramic reactivity and wettability, thus boosting chemical adhesion [34]. The application of bonding chemicals alongside SLM-fabricated Co–Cr frameworks has been shown to increase metal–ceramic bond strength, underscoring the therapeutic significance of the chemical adhesion aspect [35]. Residual stresses and localized micro-porosities, characteristic of additively created Co–Cr frameworks, may further affect oxide adhesion and diffusion during ceramic burning.

Consequently, while the current findings mainly illustrate mechanical trends, the chemical aspect of adhesion—specifically the characteristics and morphology of the oxide interface—must be considered when analyzing the lower bond strength noted in SLM specimens.

The study by Yoo et al. from 2020 [36] demonstrated that the use of bonding agents, together with sandblasting, can counteract the limitations of SLM, increasing the fragility of the interface and contributing to a mixed failure, which is clinically favorable.

Limitations include the small sample size and testing of experimental specimens without thermomechanical cycling, which may affect bond stability under clinical conditions. Future investigations with larger sample numbers are required to validate these findings. In addition, investigation of chemical bonding agents or acidic treatments (e.g., HCl) could improve the interface performance for SLM substrates.

Full SLM manufacturing parameters, such as laser power (160–195 W), scan speed (600–1200 mm/s), layer thickness (30 µm), hatch spacing (90–120 µm), and an island-scanning method with interlayer rotation, were accessible in this study. These values are within the established ideal limits for dental Co–Cr alloys and are acknowledged to affect melting pool stability, porosity development, and oxide uniformity. Their inclusion enhances the repeatability of the current findings and elucidates the processing-related methods responsible for the reduced ceramic adhesion seen in the SLM group.

The current study, focused on mechanical evaluation by a singular standardized test (ISO 9693:2012), suggests that the noted disparities are probably related to the unique microstructural properties of cast and SLM-fabricated alloys. The literature indicates that SLM surfaces have a finer grain structure, layered melt tracks, and localized porosities that affect oxide formation and ceramic wetting [18,36,37,38]. Future research will use SEM/EDS and surface roughness measurements to connect these characteristics with mechanical outcomes.

Another limitation of this study is the absence of direct visual or microscopic assessment of the fracture surfaces, which would allow a more precise correlation between the recorded force–displacement behavior and the actual failure mechanisms.

The study was limited to one mechanical testing method and did not include surface characterization or thermomechanical cycling, which could influence long-term bond performance. Therefore, the current results should be interpreted as indicative of preliminary mechanical trends rather than definitive interfacial strength, and further studies are required to validate these observations under clinically simulated aging conditions.

## 5. Conclusions

The pilot study aimed to generate hypothesis-forming data on how the manufacturing method of the metal substrate (conventional casting vs. three-dimensional printing by selective laser melting—SLM) and the sandblasting treatment with aluminum oxide of different sizes (125 μm and 250 μm) influence the adhesion strength of dental ceramics applied to CoCr alloys, by means of the standardized three-point bending test (ISO 9693:2012). The results obtained allow the formulation of the following conclusions:

1. The manufacturing method influences the adhesion of ceramics: The samples obtained by casting presented higher displacement strength values than those obtained by SLM, regardless of the sandblasting treatment applied. This result is attributed to the more compact microstructure of the cast substrates and their more predictable mechanical behavior, as shown by Yoo et al. and Hong et al. in their studies [26,37].
2. Abrasive particle size determines interface performance: Utilizing 250 μm grit blasting generated better adhesion compared to 125 μm grit treatment, both for cast and SLM samples. This effect is explained by the increase in roughness and contact area, facilitating the mechanical anchoring of the ceramic.
3. The best-performing combination: The best ceramic adhesion was recorded for the CCT_250 group (cast Co Cr, blasted with 250 μm), with an average value of 12.48 N, followed by CCT_125, CCP_250, and CCP_125. Thus, the cast substrate + 250 μm grit blasting combination is mechanically optimal for the fabrication of metal–ceramic restorations.
4. Involvement of additional treatments for SLM substrates: For alloys fabricated by SLM, the use of complementary treatments, such as bonding agents or acid pretreatment, is indicated to increase the efficiency of ceramic-to-metal bonding, as recommended in the current literature.
5. Future research directions: Further investigations are needed to evaluate the behavior of the metal–ceramic interface under clinically simulated conditions (thermomechanical cycling, accelerated aging), as well as to test the efficiency of various chemical bonding agents for SLM substrates.

The current results, limited by an exploratory pilot design and a singular standardized mechanical test, should be regarded as suggestive patterns rather than conclusive clinical forecasts. The provision of comprehensive SLM processing parameters enhances the repeatability of the current technology; nevertheless, further research is necessary to establish a direct correlation between these factors and oxide production, failure modes, and long-term interfacial stability.

## Figures and Tables

**Table 1:** Coding of samples used in the mechanical test.

Nr. Crt.	Sample Description	Coding
1	cast Co-Cr, Al_2_O_3_ particles (125 μm)	CCT_125
2	cast Co-Cr, Al_2_O_3_ particles (250 μm)	CCT_250
3	3D-printed Co-Cr, Al_2_O_3_ particles (125 μm)	CCP_125
4	3D-printed Co-Cr, Al_2_O_3_ particles (250 μm)	CCP_250

**Table 2:** Displacement forces (N) obtained from the three-point bending test for each experimental group.

Material	Test 1	Test 2	Test 3	Test 4	Test 5	Mean (N)	SD (N)
CCT_125	9.50	11.62	9.76	9.70	9.17	9.95	0.96
CCT_250	10.67	13.15	13.09	11.20	8.62	11.35	1.88
CCP_125	6.02	7.50	7.88	6.35	9.50	7.45	1.38
CCP_250	7.87	8.82	8.59	9.48	11.18	9.19	1.25

## Data Availability

The original contributions presented in this study are included in the article. Further inquiries can be directed to the corresponding authors.
